# Effect of cardiac shock wave therapy plus optimal medical therapy on rehospitalization in patients with severe coronary artery disease: A meta-analysis and trial sequential analysis

**DOI:** 10.3389/fcvm.2022.1010342

**Published:** 2022-12-12

**Authors:** Peng Li, Na Jia, Bing Liu, Qing He

**Affiliations:** ^1^The Key Laboratory of Geriatrics, Beijing Institute of Geriatrics, Institute of Geriatric Medicine, Chinese Academy of Medical Sciences, Beijing Hospital/National Center of Gerontology of National Health Commission, Beijing, China; ^2^Department of Cardiology, Beijing Hospital, National Center of Gerontology, Institute of Geriatric Medicine, Chinese Academy of Medical Sciences, Beijing, China

**Keywords:** cardiac shock wave therapy, severe coronary artery disease, major adverse cardiac events, rehospitalization, meta-analysis

## Abstract

**Objective:**

Several small sample-sized clinical studies have demonstrated that cardiac shock wave therapy (CSWT) might reduce the risk of rehospitalization in patients with severe coronary artery disease (CAD). However, other observational studies did not reported that clinical benefit of CSWT. Therefore, the effect of CSWT plus optimal medical therapy (OMT) on rehospitalization is still controversial.

**Methods:**

We performed an updated meta-analysis and systematic review of randomized clinical trials (RCTs) and prospective cohort studies identified in systematic searches of Pubmed, Embase, the Cochrane library, the ClinicalTrials.gov website and Chinese SinoMed Database (up to December 2021). Primary endpoint was the rate of major adverse cardiac events (MACEs, the composite outcome of mortality, coronary artery revascularization, and rehospitalization). Meta-regression and subgroup analyses were used to identify possible contributors to between-study variances in the HDRS. Required information size (RIS) was calculated with trial sequential analysis (TSA).

**Results:**

A total of 11 RCTs and 5 prospective cohort studies involving 1,149 patients with a mean follow-up of 10.3 months (range 3–72) months were included. Overall, CSWT plus OMT significantly decreased the rate of MACEs compared with the OMT group (RR, 0.39; 95% CI, 0.29–0.53), which was mainly attributed to markedly lower risk of rehospitalization (RR, 0.37; 95% CI, 0.27–0.51). Subgroup analysis showed that the pooled RRs for MACEs was significantly lower in studies enrolling patients with higher baseline Canadian Cardiovascular Society angina class (≥2.2) (RR, 0.36; 95% CI, 0.26–0.50) or studies with short follow-up period (followed ≤ 6 months, RR, 0.39; 95% CI, 0.24–0.64; followed 7–12 months, RR, 0.38; 95% CI, 0.26–0.54) or studies with HF with reduced ejection fraction (RR, 0.31; 95% CI, 0.13–0.72) or with preserved ejection fraction (RR, 0.40; 95% CI, 0.29–0.56). TSA showed that The RIS for MACE was 935, and the accrued information size was 577.

**Conclusion:**

Cardiac shock wave therapy plus OMT could decrease the rate of rehospitalization among patients with severe CAD. However, this result must be interpreted with caution, for the evidence supporting the use of CSWT for severe CAD is limited by the small sample size and short follow-up period of previous studies. Larger RCTs with longer follow-up are warranted to confirm these findings.

**Systematic review registration:**

[https://inplasy.com/], identifier [INPLASY202210103].

## Introduction

Severe coronary artery disease (CAD), is a disabling and prevalent condition, characterized by cardiac pain and refractory to conventional therapies including long-acting nitrates, β-receptor blockers, calcium-channel blockers, and traditional revascularization (percutaneous coronary intervention and coronary artery bypass surgery) (1, 2). The mortality rate during follow-up of these patients is 3.9% at 1 year and 28.4% at 9 years (3). Severe CAD could adversely affect the risk of mortality compared with stable, chronic CAD (4). Therefore, these individuals suffer severely impaired quality of life and increased rate of rehospitalization for cardiovascular reasons. Therapeutic strategies are thus directed primarily at improving patients’ quality of life and decreasing cardiovascular rehospitalization by relieving symptoms of angina pectoris. Although numerous innovative pharmacological (metabolic modulation and angiogenesis) and non-pharmacological (coronary sinus reducer, spinal cord stimulation, stem cell therapy, and enhanced external counterpulsation) therapeutic options have been studied recently, none have demonstrated clear clinical efficacy (5–9).

Extracorporeal cardiac shock wave therapy (CSWT) is a cutting-edge technology developed in the world for more than 20 years (10–13). Several small sample-sized clinical studies have demonstrated that CSWT might reduce the risk of rehospitalization in patients with severe CAD (14, 15). However, other observational studies did not reported that clinical benefit (16–19). Additionally, previous evidence is limited to mainly small-sized, single-arm, low- to moderate-quality, single-center studies with mixed results.

As the amount of available evidence has recently increased, we performed an updated meta-analysis and trial sequential analysis (TSA) to evaluate the effect of CSWT plus optimal medical therapy (OMT) on major adverse cardiac events (MACEs) in patients with severe CAD.

## Methods

### Data sources and search strategies

We undertook an updated meta-analysis and systematic review of randomized clinical trials (RCTs) identified in systematic searches of MEDLINE (source, PubMed from 2005 to December 2021), EMBASE (2005 to December 2021), the Cochrane Controlled Clinical Trials Register Database (to December 2021), the ClinicalTrials.gov website (to December 2021), and the Chinese SinoMed Database (to December 2021) using the terms “cardiac shock wave therapy,” “adverse cardiac event,” “rehospitalization,” “mortality,” “severe CAD,” “randomized trial,” and “prospective study.” Search Strategy: Heart Failure [OR] Coronary Artery Disease [OR] Angina [OR] (coronary* or atherosclero*) [AND] Cardiac Shockwave [OR] External Shockwave [OR] extracorporeal Shockwave [OR] Shockwave [AND] Cardiac Event [OR] Rehospitalization [OR] Revascularization [OR] Mortality.

The reference lists of all relevant articles were also manually checked. No restrictions on language were applied. The review was registered in https://inplasy.com/ (INPLASY202210103).

### Study selection

First, we performed an initial screening of titles and abstracts. Second, all articles were evaluated based on full-text review. Studies were considered eligible if they met these criteria: (1) included patients were diagnosed as severe CAD, defined as multiple or diffused coronary artery lesion and not candidates for percutaneous coronary intervention (PCI) or coronary artery bypass graft (CABG), or refractory angina not alleviated within 3 months of OMT; (2) interventions consisted of CSWT plus OMT vs. OMT alone; the study was a randomized controlled trial (RCT) or a prospective cohort study; (3) primary outcome was rate of MACE (the composite outcome of mortality, coronary artery revascularization, and rehospitalization); (4) had a RCT or prospective cohort study design.

The exclusion criteria were: (1) had heart transplant, uncontrolled heart failure, severe arrhythmia, metal valve replacement; (2) had no primary outcome; (3) was a single-arm study; (4) was a retrospective study, animal study, case report, or review; and (5) had duplicated data.

### Data extraction

Using a standard data-collection form, two reviewers (PL and NJ) extracted data concerning study’s characteristics (design, country conducted, inclusion criteria, interventions, primary and secondary outcomes, quality), patients’ characteristics (sample size, age, percentage of males, LVEF level, percentage of hypertension and diabetes, levels of Canadian Cardiovascular Society [CCS] angina class and New York Heart Association [NYHA] class, period of follow-up), interventions (target segment, intensity, times, and duration), and study endpoints. Disagreements were resolved by discussion with another reviewer (QH).

Primary outcome was the rate of MACEs (the composite outcome of mortality, coronary artery revascularization, and rehospitalization). Secondary outcomes were the rates of mortality, coronary artery revascularization and rehospitalization.

### Quality assessment

The Preferred Reporting Items for Systemic Reviews and Meta-Analyses (PRISMA) statement (20) was followed. Two reviewers (PL and BL) used the Cochrane’s risk of bias tool to evaluate the quality of included RCTs, including the generation of random sequences (selection bias), allocation concealment (selection bias), blinding of participants and personnel (performance bias), blinding of outcome assessment (detection bias), incomplete outcome data (attrition bias), selective reporting (reporting bias), and other bias. Additionally, we used the Newcastle-Ottawa scale (NOS) to evaluate the quality of included cohort studies (21). NOS scale varies from 0 to 9 stars using eight criteria that cover three components: patient selection, study groups comparability, and outcomes assessment. Studies with a NOS score of 6 and more were considered as “high quality,” while those with a score less than 6 as “low quality.”

### Data synthesis and analysis

Results were analyzed quantitatively with STATA 14.0 software (Stata Corp, College Station, TX, USA) using the fixed- and random-effects (DerSimonian and Laird random-effects) models (22). We calculated the pooled relative risk (RR) for dichotomous outcomes with 95% confidence interval (CI).

Heterogeneity was examined by the *I*^2^ statistic and the chi-squared test. A value of *I*^2^ > 50% was considered a substantial level of heterogeneity (23). For outcomes with significant heterogeneity (*I*^2^ > 50%), the random-effects models are reported in the text and figures; for all others, the fixed-effects models are reported. Once heterogeneity was noted, between-study sources of heterogeneity were investigated using subgroup analysis by stratifying original estimates according to study characteristics. Publication bias was assessed quantitatively using the Begg’s adjusted rank correlation test or the Egger’s regression asymmetry test (*P* ≤ 0.10 for both) (24). Sensitivity analyses were conducted to determine the influence of individual trials on the overall pooled results (MACEs, rehospitalization). All analyses were performed according to the intention-to-treat principle. Statistical significance was set at 0.05.

### Meta-regression

Univariate meta-regression analysis was used to identify possible contributors to between-study variance. We investigated the associations between the RRs for MACEs, rehospitalization and clinically plausible factors, including age, male, LVEF, diabetes, hypertension, CCS angina class, NYHA class, study design, and duration of follow-up.

### Subgroup analysis

Rates of MACEs and rehospitalization were also evaluated by subgroup analysis. Based on the type of design (RCT vs. Cohort study), the studies were divided into “RCTs” and “cohort studies” subgroups. In accordance with mean value of baseline clinical factors, all studies were classified into subgroups based on age (<65.2 years or ≥65.2 years), proportion of males (<75.4% or ≥75.4%), percentage of patients with hypertension (<72.3% or ≥72.3%), percentage of patients with diabetes mellitus (<41.4% or ≥41.4%), CCS angina class (<2.2 or ≥2.2), NYHA class (<2.2 or ≥2.2), countries that studies conducted (China or other countries). On the basis of follow-up duration, studies were divided into subgroups of ≤6 months, 7–12 months, and > 12 months. In addition, according to the classification of heart failure, studies were also divided into subgroups of heart failure with reduced ejection fraction (LVEF < 40%), heart failure with mid-range ejection fraction (LVEF 40–49%) and heart failure with preserved ejection fraction (LVEF ≥ 50%).

### Trial sequential analysis

In this meta-analysis, TSA was used to reduce the risk of reaching a false-positive or false-negative conclusion (25). When the cumulative Z-curve crossed the trial sequential monitoring boundary or entered the futility area, a sufficient level of evidence for the anticipated intervention effect was reached, and no further trials were needed. If the Z-curve did not cross any of the boundaries and the required information size (RIS) had not been reached, the evidence was deemed insufficient to reach a conclusion, and more trials were needed to confirm the results. For this TSA for MACEs and rehospitalization, we estimated the RIS based on a RR reduction of 20%. The type I error (α) = 0.05 (two-sided) and power (1–β) = 0.80. The control event proportions were 45% for MACEs and 35% for rehospitalization, respectively. The TSA was conducted using TSA software, version 0.9.5.10 Beta.^1^

## Results

### Search results

**FIGURE 1:**
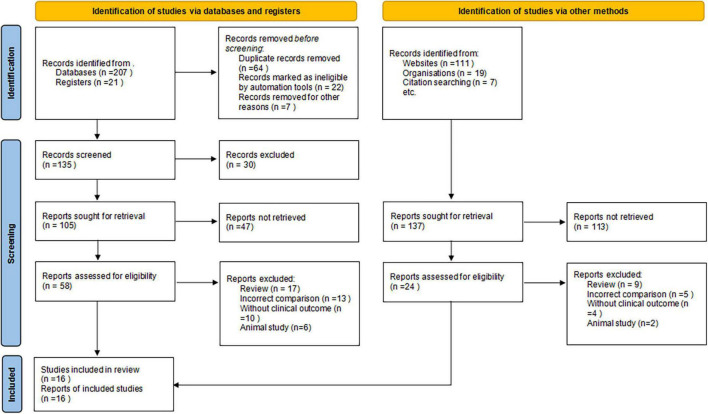
Flow chart of study selection.

We initially identified 365 potentially relevant articles. Eighty-four papers were considered to be of interest and were retrieved for full-text review. Sixty-six articles were excluded for reasons of reviews (*n* = 26), incorrect comparisons (*n* = 18), no clinical outcomes (*n* = 14), and animal study (*n* = 8). The remaining 11 RCTs and 5 prospective cohort studies were finally included in the analysis (Figure 1).

### Study characteristics

A total of 11 published RCTs (26–36) and 5 cohort studies (17, 37–40) involving a total of 1,149 patients with severe CAD were included. The total number of patients in each study was in the range of 25–150, and the median follow-up period was 10.3 months (range 3–72 months). The average participants’ age was 65.2 years. Approximately 75.4% of patients were men and nearly half of the patients (41.4%) had diabetes. Most patients enrolled had HF with preserved ejection fraction (LVEF ≥ 50%). The average CCS angina class 2.2 and NYHA class was 2.2 ± 0.5. There were three comparisons in two studies (26, 30).

Based on the CSWT treatment scope for each ischemic target region, patients in the one study (38) were divided into the regular CSWT group (9 spots of each ischemic target region, performed 9 times within 3 months), expanding scope CSWT group (25 spots of each ischemic target region, performed 9 times within 3 months), and the control group. Moreover, patients in one study (26) were separated into a regular CSWT group (performed 9 times within 3 months), a short-term CSWT group (performed 9 times within 1 month), and a control group according to the CSWT treatment duration. A short-term CSWT treatment procedure (9 times within 1 month) was also used in another study (17; Table 1).

**TABLE 1:** Baseline characteristics of patients in the CSWT plus OMT group and OMT group.

First author	Year	Patients	Num.	Age, year	Male, %	LVEF, %	HP, %	DM, %	CCS angina class	NYHA class	Follow, m
Wang et al. (26)§	2012	RA	20/21/14	62.7/64.1/67.9	90.0/81.0/85.7	NR	20.0/19.0/42.9	35.0/19.0/28.6	2.0/3.0/2.0	1.5/2.0/2.0	12
Peng et al. (27)§	2012	CAD + CHF	25/25	66.0/67.0	68.0/72.0	45.0/46.0	44.0/40.0	64.0/68.0	2.0/2.0	2.0/2.0	6
Kazmi et al. (37)#	2012	CAD	43/43	58.7/56.6	87.0/84.0	49.6/43.6	56.0/44.0	77.0/74.0	NR	2.5/2.5	6
Yang et al. (28)§	2012	CAD	25/20	67.5/66.1	76.0/68.0	34.0/30.0	72.0/75.0	44.0/40.0	2.7/2.6	2.2/2.3	6
Yang et al. (29)§	2013	CAD	14/11	63.7/66.5	71.4/72.7	51.4/50.2	57.1/45.5	42.9/45.5	2.0/2.0	2.0/1.0	8.4
Zhao et al. (30)§	2015	RA	32/30/25	68.3/65.7/66.2	75.0/80.0/80.0	NR	65.6/60.0/64.0	40.6/50.0/44.0	2.6/2.6/2.5	2.2/2.1/2.2	12
Alunni et al. (38)#	2015	RA	43/29	70.0/71.0	83.7/79.0	56.4/57.3	100/100	32.5/27.0	2.8/2.5	2.5/2.3	12
Nirala et al. (17)#	2016	CAD	41/11	63.4/71.0	85.4/72.7	NR	65.9/54.5	26.8/45.5	2.2/1.8	1.9/1.4	72
Song et al. (31)§	2016	CAD + CHF	28/25	67.0/66.0	78.5/68.0	37.4/38.3	64.0/54.0	43.0/44.0	2.8/2.7	2.8/2.7	3
Liu et al. (32)§	2017	RA	36/9	70.5/65.9	69.0/67.0	NR	58.0/56.0	NR	1.9/1.4	NR	3
Shkolnik et al. (33)	2018	CAD	37/35	67.6/68.8	62.2/82.8	54.5/56.5	96.3/97.1	21.6/28.6	NR	NR	6
Zhang et al. (39)#§	2018	CAD	90/90	62.5/61.3	53.3/57.8	44.1/44.1	NR	NR	2.6/2.4	2.4/2.1	3
Čelutkienë et al. (34)	2019	CAD	30/29	67.2/69.4	63.3/89.7	54.4/56.0	96.7/100	26.7/27.6	NR	NR	6
Alunni et al. (40)#	2020	RA	121/29	70.0/71.0	79.0/79.0	56.5/57.3	98.0/100	33.0/27.0	2.7/2.5	2.5/2.3	6
Jia et al. (35)§	2021	RA	15/15	69.2/71.4	66.7/73.3	62.5/63.0	86.7/93.3	73.3/53.3	NR	NR	3
Weijing et al. (36)§	2021	RA	46/41	68.1/68.9	70.0/71.0	48.8/47.8	59/56	52/56	2.9/2.8	NR	6

§, study conducted in China; #, prospective cohort study; RA, refractory angina; CAD, coronary artery disease; CHF, chronic heart failure; Num., number; LVEF, left ventricular ejection fraction; HP, hypertension; DM, diabetes mellitus; CCS, Canadian Cardiovascular Society; NYHA, New York Heart Association; m, month; NR, not reported.

### Methodological quality assessment

**FIGURE 2:**
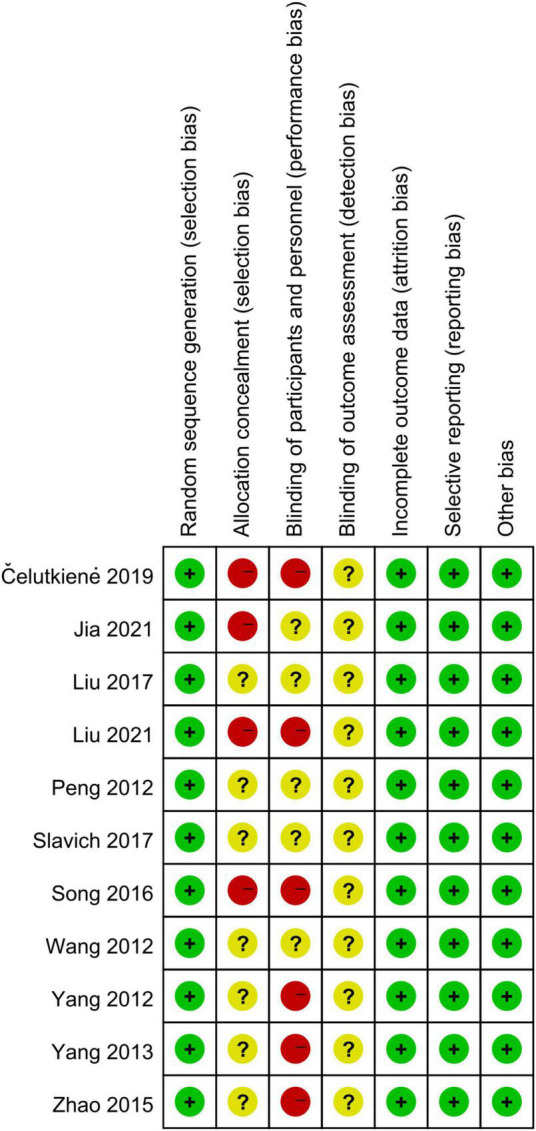
Quality evaluation with Cochrane’s risk of bias tool.

Eleven RCTs randomized the participants. However, one RCT did not report the methodological details of random sequence generation (29). Six RCT studies used satisfactory methods of treatment allocation concealment (15–17, 28–30, 37). Blinding of participants and personnel was reported in five studies (15–17, 28, 37). There was a low risk of attrition bias and reporting bias in all studies (Figure 2).

Most cohort studies (17, 37–39) had a NOS score of 7, and another study (40) had 8. Thus, all cohort studies were with a NOS score of 6 and more and were considered as “high quality.”

### Primary endpoint

#### Major adverse cardiac events

**FIGURE 3:**
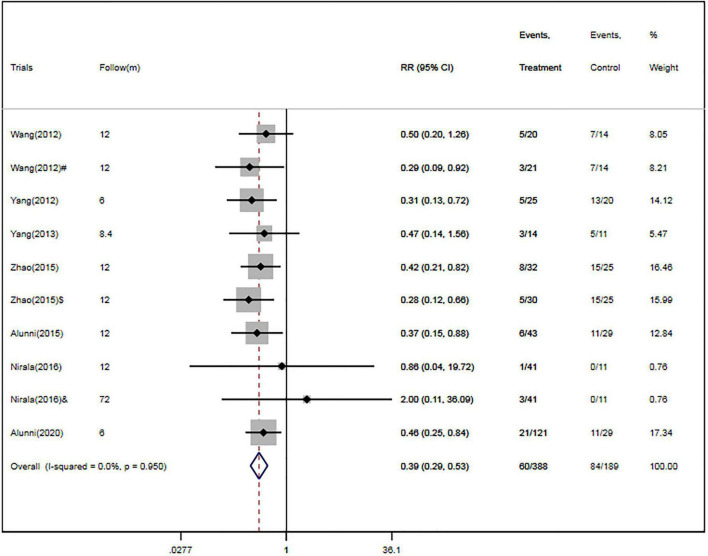
CSWT plus OMT is associated with a decreased risk of MACEs. Fixed-effects model (*I*^2^ = 0.0%). CSWT, cardiac shock wave therapy; OMT, optimal medical therapy; MACE, major adverse cardiac event; RR, relative risk; CI, confidence interval. ^#^Patients treated with short-term regimen of CSWT; ^$^, patients treated with an expanding scope CSWT; and ^&^, patients with long-term follow-up.

**FIGURE 4:**
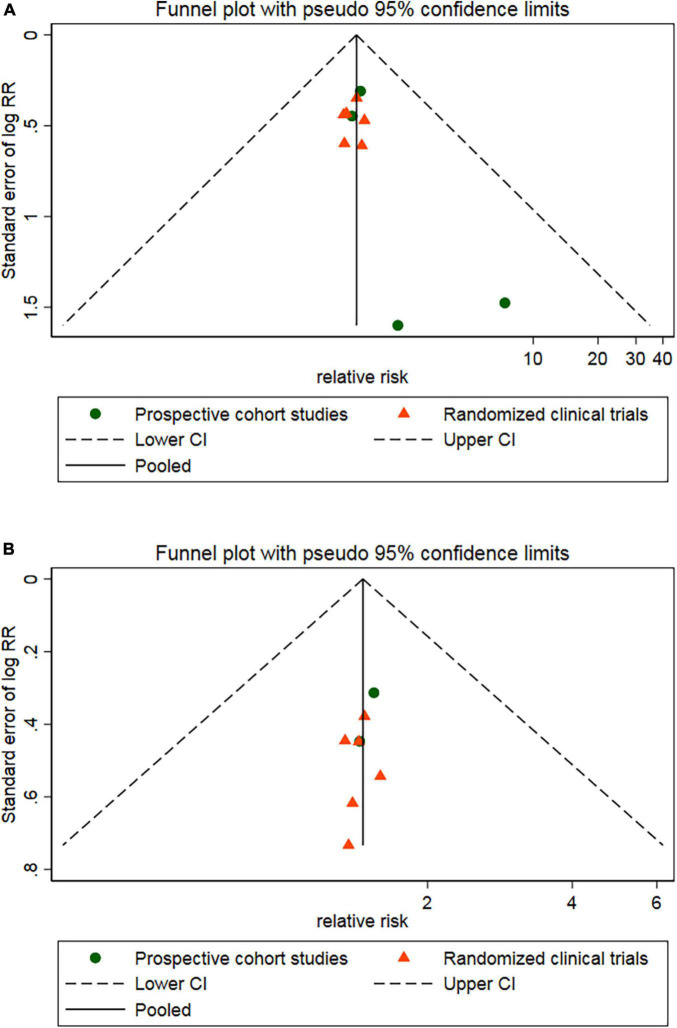
Egger’s funnel plot indicated low level of heterogeneity for evaluating MACEs **(A)** and rehospitalization **(B)**. MACE, major adverse cardiac event; RR, relative risk; CI, confidence interval.

Four RCTs (26, 28–30) and three cohort studies (17, 38, 40) provided data about MACE. Of the 388 patients in the CSWT plus OMT group, MACEs occurred in 60 patients (15.5%). In the OMT group, MACEs occurred in 84 patients out of a total of 189 (44.4%). Compared with the OMT group, CSWT plus OMT significantly lowered the risk of MACEs (RR, 0.39; 95% CI, 0.29–0.53; *P* < 0.001) (Figure 3), and there was low level of heterogeneity (*I*^2^ = 0.0%). The funnel plot did not show marked asymmetry in Begg’s test (*P* = 0.67) or Egger’s test (*P* = 0.82) (Figure 4A). Sensitivity analysis was performed by removing each of the trials one at a time, which did not detect any influence on the risk of MACEs (Table 2).

**TABLE 2:** Outcomes of the CSWT plus OMT group and OMT group.

Outcomes	Num. of event in CSWT plus OMT group	Num. of event in OMT group	RR (95% CI)	*P*	*I* ^2^
**Primary outcome**					
MACEs	60/388	84/189	0.39 (0.29–0.53)	<0.001	0
**Secondary outcomes**					
Rehospitalization	52/306	78/167	0.37 (0.27–0.51)	<0.001	0
Revascularization	1/187	3/77	0.31 (0.07–1.44)	0.136	0
All-cause mortality	7/203	3/117	0.93 (0.32–2.65)	0.887	0

MACE, major adverse cardiac event; CSWT, cardiac shock wave therapy; OMT, optimal medical therapy; RR, relative risk; CI, confidence interval.

### Secondary endpoints

#### Rehospitalization

**FIGURE 5:**
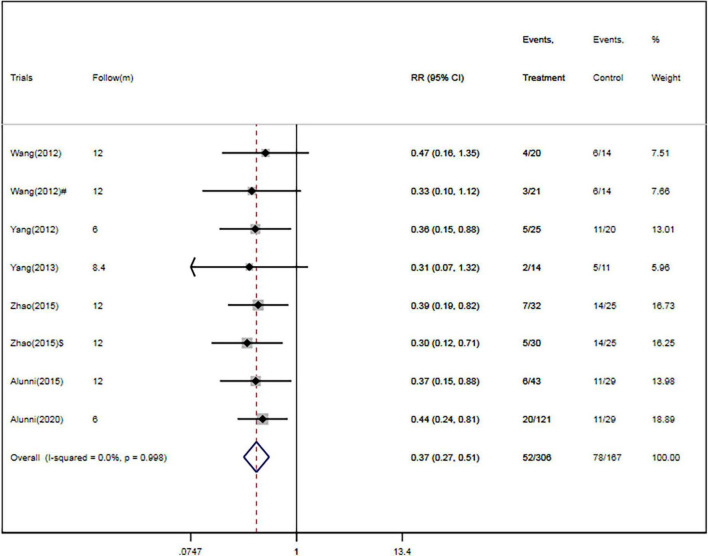
CSWT plus OMT is associated with a decreased rate of rehospitalization. Fixed-effects model (*I*^2^ = 0.0%). CSWT, cardiac shock wave therapy; OMT, optimal medical therapy; RR, relative risk; CI, confidence interval. ^#^Patients treated with short-term regimen of CSWT; ^$^, patients treated with an expanding scope CSWT.

Four RCTs (26, 28–30) and two cohort studies (38, 40) reported the occurrence of rehospitalization. There were 306 patients in the CSWT plus OMT group, 52 of whom were rehospitalized (17.0%). Of the 167 patients in the OMT group, 78 were rehospitalized (46.7%). Overall, CSWT plus OMT was associated with a significantly decreased rate of rehospitalization (RR, 0.37; 95% CI, 0.27–0.51; *P* < 0.001) compared with the OMT group (Figure 5). In addition, there was a low level of heterogeneity (*I*^2^ = 0.0%), and the funnel plot did not show marked asymmetry in Begg’s test (*P* = 0.71) or Egger’s test (*P* = 0.76) (Figure 4B and Table 2).

### Coronary artery revascularization

The rate of coronary artery revascularization during the follow-up period was presented in two RCTs (26, 28) and one cohort study (35). There were one (0.5%) and three cases (3.9%) of coronary artery revascularization in the CSWT plus OMT (*n* = 187) and OMT (*n* = 77) groups, respectively. Overall, the rate of revascularization was not significantly different between the CSWT plus OMT and OMT groups (RR, 0.31; 95% CI, 0.07–1.44; *P* = 0.136). Moreover, there was a low level of heterogeneity (*I*^2^ = 0.0%), and the funnel plot did not show marked asymmetry in Begg’s test (*P* = 0.74) or Egger’s test (*P* = 0.83) (Table 2).

### All-cause mortality

The risk of all-cause mortality was specified in four RCTs (26, 28–30) and one cohort study (17). Seven out of 203 patients (3.4%) in the CSWT plus OMT group and 3 out of 117 (2.6%) in the OMT group died. Overall, CSWT plus OMT was associated with a risk of mortality similar to that in the OMT group (RR, 0.93; 95% CI, 0.32–2.65; *P* = 0.887). There was a low level of heterogeneity (*I*^2^ = 0.0%), and the funnel plot did not show marked asymmetry in Begg’s test (*P* = 0.73) or Egger’s test (*P* = 0.69) (Table 2).

### Sensitivity analysis

**FIGURE 6:**
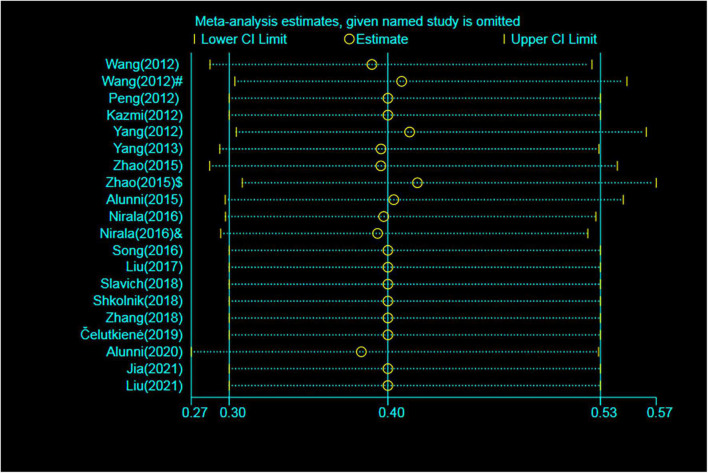
Sensitivity analysis for MACEs. MACE, major adverse cardiac event; RR, relative risk; CI, confidence interval. ^#^Patients treated with short-term regimen of CSWT; ^$^, patients treated with an expanding scope CSWT; and ^&^, patients with long-term follow-up.

To determine the influence of individual trials on the overall pooled results of MACEs and rehospitalization, we performed the sensitivity analysis by removing each of the trials one at a time, which did not detect any influence on the overall result of MACEs or rehospitalization (*P* > 0.05) (Figure 6).

### Meta-regression analyses

In meta-regression, no significant correlations were observed between the RRs for MACEs and study design (*t* = 0.34, *p* = 0.74), age (*t* = 0.08, *p* = 0.94), male (*t* = 1.05, *p* = 0.31), LVEF (*t* = 0.24, *p* = 0.83), hypertension (*t* = −0.02, *p* = 0.97), diabetes (*t* = −0.17, *p* = 0.87), CCS angina class (*t* = −0.24, *p* = 0.82), NYHA class (*t* = −0.37, *p* = 0.73), follow-up duration (*t* = 0.95, *p* = 0.37) and country (*t* = 0.35, *p* = 0.73).

Additionally, study design (*t* = 0.15, *p* = 0.89), age (*t* = 0.15, *p* = 0.89), male (*t* = 0.11, *p* = 0.92), LVEF (*t* = 0.10, *p* = 0.93), hypertension (*t* = 0.08, *p* = 0.94), diabetes (*t* = −0.18, *p* = 0.86), CCS angina class (*t* = −0.04, *p* = 0.97), NYHA class (*t* = 0.05, *p* = 0.96), follow-up duration (*t* = −0.12, *p* = 0.91) and country (*t* = 0.27, *p* = 0.79) were not significantly associated with the pooled RRs for rehospitalization.

### Subgroup analysis

**FIGURE 7:**
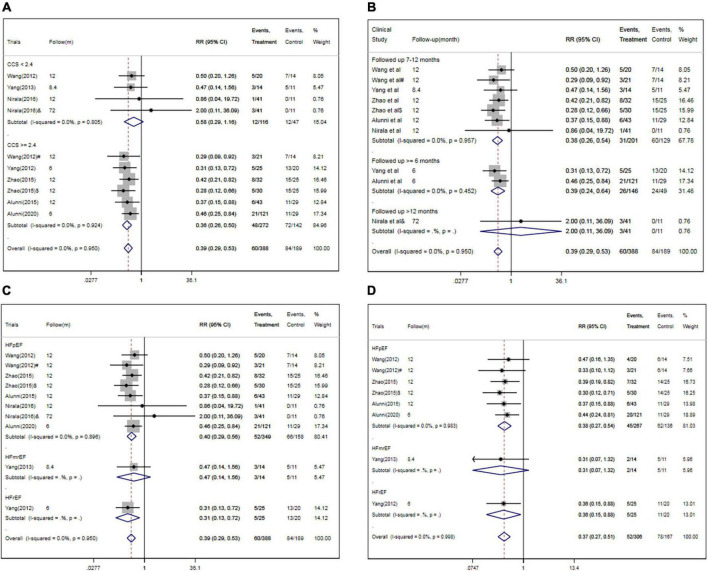
Subgroup analysis for MACEs and/or rehospitalization in studies specified by baseline CCS angina class **(A)**, follow-up period **(B)**, HF classification **(C,D)**. CSWT, cardiac shock wave therapy; MACE, major adverse cardiac event; RR, relative risk; CI, confidence interval; CCS, Canadian Cardiology Society. ^#^Patients treated with short-term regimen of CSWT; ^$^, patients treated with an expanding scope CSWT; and ^&^, patients with long-term follow-up.

In subgroup analysis, the pooled RRs for MACEs were significantly decreased in studies enrolling patients with higher CCS angina class (CCS ≥ 2.2) (RR, 0.36; 95% CI, 0.26–0.50; *P* < 0.001) (Figure 7A) or studies with short follow-up period (followed ≤ 6 months, RR, 0.39; 95% CI, 0.24–0.64; followed 7–12 months, RR, 0.38; 95% CI, 0.26–0.54) (Figure 7B) or studies with HF with reduced ejection fraction (RR, 0.31; 95% CI, 0.13–0.72) or with preserved ejection fraction (RR, 0.40; 95% CI, 0.29–0.56) (Figure 7C). However, there were no significant differences between other subgroups in pooled RRs for MACEs (Table 3).

**TABLE 3:** Subgroup analyses for MACE and rehospitalization.

Variables		MACE				Rehospitalization for HF worsening			
	Subgroups	Patient’s num.	RR (95% CI)	*P*	*I* ^2^	Patient’s num.	RR (95% CI)	*P*	*I* ^2^
Study design	RCT	251	0.36 (0.25–0.52)	0.000	0	251	0.36 (0.24–0.53)	0.000	0
	Cohort study	326	0.47 (0.29–0.76)	0.000	0	222	0.41 (0.24–0.68)	0.001	0
Age (year)	<65.2	355	0.38 (0.27–0.55)	0.000	0	251	0.36 (0.24–0.53)	0.000	0
	>65.2	222	0.42 (0.25–0.69)	0.001	0	222	0.41 (0.24–0.68)	0.000	0
Male (%)	<75.4	70	0.35 (0.18–0.70)	0.000	0	70	0.35 (0.16–0.74)	0.000	0
	>75.4	507	0.40 (0.29–0.56)	0.000	0	403	0.38 (0.27–0.54)	0.000	0
HF (%)	HFrEF	45	0.31 (0.13–0.72)	0.006	0	45	0.36 (0.15–0.88)	0.024	0
	HFmrEF	25	0.47 (0.14–1.56)	0.217	0	25	0.31 (0.07–1.32)	0.114	0
	HFpEF	507	0.40 (0.29–0.56)	0.000	0	403	0.38 (0.27–0.54)	0.000	0
HP (%)	<72.3	310	0.40 (0.27–0.60)	0.000	0	206	0.36 (0.23–0.55)	0.000	0
	>72.3	267	0.38 (0.25–0.58)	0.001	0	267	0.39 (0.25–0.61)	0.000	0
DM (%)	<44.2	497	0.41 (0.30–0.57)	0.000	0	393	0.39 (0.28–0.56)	0.000	0
	>44.2	55	0.28 (0.12–0.66)	0.002	0	80	0.30 (0.14–0.64)	0.002	0
CCS angina class	<2.2	59	0.58 (0.29–1.16)	0.124	0	59	0.40 (0.17–0.94)	0.035	0
	>2.2	518	0.36 (0.26–0.50)	0.000	0	414	0.37 (0.26–0.52)	0.000	0
NYHA class	<2.2	198	0.48 (0.27–0.86)	0.014	0	94	0.38 (0.19–0.75)	0.006	0
	>2.2	379	0.37 (0.26–0.52)	0.000	0	379	0.37 (0.26–0.53)	0.000	0
Follow–up duration (m)	≤6	195	0.39 (0.24–0.64)	0.000	0	195	0.41 (0.24–0.67)	0.000	0
	7–12	330	0.38 (0.26–0.54)	0.000	0	278	0.36 (0.24–0.53)	0.000	0
	>12	52	2.00 (0.11–36.09)	0.639	0	NR			
Country	China	251	0.36 (0.25–0.52)	0.000	0	251	0.36 (0.24–0.53)	0.000	0
	Other countries	326	0.47 (0.29–0.75)	0.002	0	222	0.41 (0.24–0.68)	0.001	0

MACE, major adverse cardiac event; RCT, randomized controlled trial; LVEF, left ventricular ejection fraction; HP, hypertension; DM, diabetes mellitus; CCS, Canadian Cardiology Society; NYHA, New York Heart Association; Num., number; RR, relative risk; NR, not reported.

Meanwhile, the pooled RRs for rehospitalization was significantly decreased in studies enrolling patients with HF with reduced ejection fraction (RR, 0.36; 95% CI, 0.15–0.88) or with preserved ejection fraction (RR, 0.38; 95% CI, 0.27–0.54) (Figure 7D). However, there were no significant differences between other subgroups in pooled RRs for rehospitalization (Table 3).

### Trial sequential analysis

Assuming a 20% difference between CSWT plus OMT and OMT groups in the risk of MACEs, TSA showed that the RIS was 935 participants. The cumulative Z-curve crossed the trial sequential boundary, indicating a statistically significant difference in the risk of MACEs between the group that underwent CSWT plus OMT treatment and the group that underwent OMT alone.

In addition, assuming a 20% difference between CSWT plus OMT and OMT groups in the risk of rehospitalization, TSA showed that the RIS was 1,383 participants. The cumulative Z-curve crossed the trial sequential boundary, indicating a lower risk of rehospitalization with CSWT plus OMT treatment than with OMT among severe CAD patients.

## Discussion

This up-to-date meta-analysis of the available evidence showed that CSWT plus OMT significantly reduced the rate of rehospitalization (RR, 0.37; 95% CI, 0.27–0.51) and MACE (RR, 0.39; 95% CI, 0.29–0.53) in patients with severe CAD. Subgroup analysis showed that the pooled RRs was significantly lower in studies enrolling patients with higher CCS angina class (≥2.4) (RR, 0.36; 95% CI, 0.26–0.50). TSA showed that the RIS for MACEs was 935, and the accrued information size was 577. One could conclude that CSWT can offer beneficial effects to patients with severe CAD.

Cardiac shock wave therapy plus OMT can potentially reduce the rate of rehospitalization in patients with severe CAD. The treatment of severe CAD is challenging, as patients with severe CAD experience angina even with minimal activity or at rest (41, 42). Therefore, these individuals suffer a severely increased rate of rehospitalization for frequent angina, although the risk of mortality is similar to that for stable CAD (4). CSWT has been reported to potentially promote coronary angiogenesis in ischemic myocardium (11), inhibit ischemia/hypoxia-induced H9c2 myoblast cell apoptosis (14), promote cardiomyocyte autophagy during hypoxia (15), improve myocardial blood flow (12), reduce angina symptoms (13), and increase cardiac function (43). Hence, CSWT may potentially decrease the rate of rehospitalization as a consequence of angina symptoms. In a clinical study of CSWT for 45 CAD patients, Yang et al. found that CSWT markedly decreased the rate of rehospitalization for myocardial ischemic symptoms at 6-month follow-up in comparison with a control group (20.0% vs. 55.0%, *P* < 0.05) (28). In another study conducted in China with 12 months of follow-up, old myocardial infarction patients in the control group were associated with a significantly higher rate of rehospitalization because of CAD when compared with patients in the regular CSWT group (56.0 vs. 21.8%) or those in the expanding scope CSWT group (56.0 vs. 16.7%) (31). Consistent with previous studies, this meta-analysis confirms the clinical benefit of CSWT with respect to rehospitalization for frequent angina. However, two studies demonstrated that CSWT, whether with a regular (performed 9 times within 3 months) or short-term (performed 9 times within 1 month) pattern (26, 30), was unable to decrease the risk of rehospitalization within 8–12 months of follow-up. All included studies are limited to single-center, mostly uncontrolled and underpowered trials, and no study evaluated the long-term effect of CSWT for severe CAD. Therefore, more studies are needed to confirm the potential clinical benefit of CSWT.

Cardiac shock wave therapy produces a prominent anti-ischemic effect for patients with severe CAD. Several studies have confirmed that CSWT was associated with significantly improved myocardial perfusion, cardiac function, exercise tolerance, myocardial ischemia symptoms, and quality of life in patients with severe CAD over short-term (26, 30, 33–36, 38–41) and long-term (17) follow-up. In a multicenter trial involving 50 severe CAD patients from four institutes in Japan (11), Kikuchi et al. demonstrated that CSWT markedly improved the angina symptoms and 6-min walking distance. However, the percent myocardial ischemia assessed by drug-induced stress myocardial perfusion imaging tended to be improved only in the treated segments (*P* = 0.06), and no change was noted in the whole left ventricle (11). As previous studies are limited to single-center, mostly uncontrolled and underpowered trials, most publications on CSWT provide only low- to moderate-quality results on the clinical benefits of CSWT. Recently in a prospective, randomized, triple-blind, sham-procedure-controlled study, 72 severe CAD patients were randomized at a 1:1 ratio to an optimal medical therapy plus CSWT group (*n* = 37) and an optimal medical therapy with sham-procedure group (*n* = 35), whereby at 6-month follow-up CSWT exerted a neutral effect on quality of life and level of angina. Moreover, exercise duration in the modified Bruch treadmill test was not significantly improved with CSWT (*P* > 0.05) (39), which was consistent with the findings of a study by Leibowitz et al. (44). The less pronounced effect of CSWT might be attributed to the different protocol used. In contrast to previous studies that applied CSWT only to ischemic segments, this study provided CSWT sequentially to all segments of the left ventricle. In addition, placebo may have a significant ameliorating effect on subjective outcome assessments such as angina (34), SAQ score, and exercise capacity, and the PACIFIC trial found that 28% of improvement in the CCS angina class was due to investigator bias (45). Thus, as both investigators and patients tend to be biased toward an improvement over time because of placebo, a modest CSWT effect in this study (39) might be masked by a prominent placebo effect. Furthermore, exercise duration is insufficiently sensitive and specific for ischemic assessment, which could be better evaluated by stress echocardiography of single-photon emission computed tomography to estimate the anti-ischemic effect of CSWT.

Recently, several updated meta-analyses of CSWT in patients with severe CAD has been published (18, 19, 46, 47). Compared with this analyses, our study has provided several new findings. First, all studies included in our analysis are RCTs or prospective cohort studies, whereas single-arm and retrospective studies are also included in the two previous meta-analyses (18, 19, 46, 47), which could increase the risk of bias. Furthermore, the effects of CSWT plus OMT on MACEs were rarely reported in recent meta-analyses. Our study indicates that CSWT plus OMT could significantly decrease the risk of rehospitalization in patients with severe CAD, although more studies are needed to confirm these findings.

### Study limitations

The present study has a few limitations that should be noted. (1) Our analysis is based on study-level data, and it is possible that there are flaws in the original studies. (2) All studies included are single-center, uncontrolled, and underpowered trials, which may be increase the risk of bias and lower the methodological quality. (3) Our analysis enrolled patients with different CSWT protocols, which might be associated with different anti-ischemic effects. (4) The sample size of this meta-analysis was inadequate to exclude small differences in outcome between the two groups. TSA showed that the RIS for MACEs and rehospitalization were 935 and 1,383, respectively. However, the accrued information size were 577 and 473, respectively. (5) The average follow-up duration was limited to 6.9 months. The benefit of CSWT plus OMT on hard endpoint (MACEs, rehospitalization) is expected to increase over time; thus, larger RCTs with longer follow-up period are needed to definitively address this issue. Therefore, our meta-analysis represents just a possible indication, and future RCTs will require larger numbers of patients, careful matching of key clinical and technical variables, and a longer follow-up to definitively quantify the potential clinical benefit of CSWT plus OMT on hard endpoint (MACEs, rehospitalization) among severe CAD patients.

## Conclusion

Our meta-analysis indicates that CSWT plus OMT could effectively decrease the rate of rehospitalization and MACEs in patients with severe CAD. However, TSA shows that the RIS for MACEs is 935, and the accrued information size is 577. However, this result must be interpreted with caution, for the evidence supporting the use of CSWT plus OMT for severe CAD is limited by the small sample size and short follow-up period of previous studies. Larger RCTs with longer follow-up are warranted to confirm the clinical benefit on hard endpoint.

## Data availability statement

The raw data supporting the conclusions of this article will be made available from the corresponding author by request.

## Author contributions

PL wrote the main manuscript text. PL, NJ, and BL analyzed the data. QH designed the study. All authors contributed to the article and approved the submitted version.

## Footnotes

1
www.ctu.dk/tsa
